# Tyrosine Phosphorylation Based Homo-dimerization of *Arabidopsis* RACK1A Proteins Regulates Oxidative Stress Signaling Pathways in Yeast

**DOI:** 10.3389/fpls.2016.00176

**Published:** 2016-02-24

**Authors:** Mercy Sabila, Nabanita Kundu, Deana Smalls, Hemayet Ullah

**Affiliations:** Department of Biology, Howard University, WashingtonDC, USA

**Keywords:** RACK1A, *Arabidopsis*, yeast, oxidative stress, UV-B, split-ubiquitin, homo-dimerization

## Abstract

Scaffold proteins are known as important cellular regulators that can interact with multiple proteins to modulate diverse signal transduction pathways. RACK1 (Receptor for Activated C Kinase 1) is a WD-40 type scaffold protein, conserved in eukaryotes, from *Chlamydymonas* to plants and humans, plays regulatory roles in diverse signal transduction and stress response pathways. RACK1 in humans has been implicated in myriads of neuropathological diseases including Alzheimer and alcohol addictions. Model plant *Arabidopsis thaliana* genome maintains three different *RACK1* genes termed *RACK1A, RACK1B*, and *RACK1C* with a very high (85–93%) sequence identity among them. Loss of function mutation in *Arabidopsis* indicates that RACK1 proteins regulate diverse environmental stress signaling pathways including drought and salt stress resistance pathway. Recently deduced crystal structure of *Arabidopsis* RACK1A- very first among all of the RACK1 proteins, indicates that it can potentially be regulated by post-translational modifications, like tyrosine phosphorylations and sumoylation at key residues. Here we show evidence that RACK1A proteins, depending on diverse environmental stresses, are tyrosine phosphorylated. Utilizing site-directed mutagenesis of key tyrosine residues, it is found that tyrosine phosphorylation can potentially dictate the homo-dimerization of RACK1A proteins. The homo-dimerized RACK1A proteins play a role in providing UV-B induced oxidative stress resistance. It is proposed that RACK1A proteins ability to function as scaffold protein may potentially be regulated by the homo-dimerized RACK1A proteins to mediate diverse stress signaling pathways.

## Introduction

A number of environmental conditions including drought, cold, high heat, salt, UV light, water stress, and oxygen deprivation (hypoxia and anoxia) are some common adverse environmental stresses encountered by land plants. To cope with these environmental stresses, plants often utilize a variety of overlapping physiological and metabolic response pathways (Zhu, 2002; Rizhsky et al., 2004). In this regard, scaffold proteins are uniquely poised to integrate signals from multiple pathways by bringing interacting signaling components to proximity (Dard and Peter, 2006). Such proteins generate considerable functional diversity by mediating concomitant and/or promiscuous interactions with a vast array of protein partners.

WD-40 repeat containing scaffold protein RACK1 (Receptor for Activating C Kinase 1) in metazoans plays a major role in coordinating different signal transduction pathways ranging from cell division to ion channel regulation by interacting with diverse proteins (McCahill et al., 2002; Sklan et al., 2006; Chen et al., 2008; Adams et al., 2011). The recent identification that RACK1 is a core component of the eukaryotic 40S ribosomal subunit suggests that its signaling functions might directly influence the efficiency and specificity of translation as well (Link et al., 1999; Ji et al., 2004; Coyle et al., 2008; Yatime et al., 2011). RACK1 has been found to regulate miRNA pathway- indicating its diverse role in modulating developmental pathways as well (Jannot et al., 2011; Speth et al., 2013; Chu et al., 2014). The protein was identified through its ability to function as a scaffold protein, stabilizing signaling complexes involving protein kinase C (Mochly-Rosen et al., 1995). RACK1 proteins with seven WD-40 repeats are highly conserved (70–80% at the protein level) in wide range of species, including plants, humans, rats, chickens, flies, nematodes, algae, and yeasts.

As opposed to a single gene in metazoans, model plant *Arabidopsis thaliana* genome maintains three different *RACK1* genes – termed *RACK1A, RACK1B*, and *RACK1C*. These *Arabidopsis* genes- without providing transcriptional compensations- are found to regulate plant development with unequal genetic redundancy (Guo and Chen, 2008). Double and triple mutation in *Arabidopsis RACK1* genes revealed that the difference in gene expression level and the cross-regulation may determine the role played by the individual genes in regulating plant development (Guo and Chen, 2008). *Arabidopsis RACK1A* mediates multiple hormonal, developmental, and environmental conditions like drought stress signaling pathways (Chen et al., 2006; Ullah et al., 2008; Fennell et al., 2012; Kundu et al., 2013).

So far the Biomolecular Interaction Network Database reports that metazoan RACK1 interacts with more than 90 different proteins ranging from ion channels to diverse ribosomal proteins (Bader et al., 2003). A recent split-ubiquitin based *Arabidopsis* inflorescence cDNA library screen revealed that the *Arabidopsis* RACK1A protein interacts with nearly 100 different proteins (Kundu et al., 2013). Interestingly, 31% of these interacting proteins were diverse stress responsive proteins, suggesting a connection of RACK1A with the plant stress response pathway.

Unlike animal RACK1 which is encoded by a single copy gene in the respective genomes, all reported plant RACK1 genes are found to be a member of multi-gene family, possibly an effect of whole genome duplication. The presence of more than one copy of RACK1 in most plant species provides multiple opportunities for RACK1 based protein–protein interaction signaling modules (Kundu et al., 2013). Despite the functional conservation of RACK1 mediated protein–protein interaction regulated signaling modes in eukaryotes, the structural basis of such interactions are largely unknown. The deduced crystal structure of the predominant RACK1A from *A. thaliana*, provides an opportunity to elucidate the structural basis of such interactions (Ullah et al., 2008). In addition to revealing the surface residues capable of mediating protein–protein interactions, the structure also revealed the likely sites of putative post-translational modifications like phosphorylation and sumoylation of key residues (Ullah et al., 2008).

Previous reports indicated that RACK1 can dimerize *in vivo* and that this dimerization is required for specific processes including the regulation of the *N*-methyl-D-aspartate (NMDA) (Thornton et al., 2004) and the oxygen-independent degradation of hypoxia-inducible factor (HIF-1) (Liu et al., 2007). Yatime et al. (2011) reported the crystal structure of the yeast RACK1 dimer showing a unique mode of dimerization where the propeller structure is completely reorganized around blade 4 to allow inter-twining of two monomers. Dimerization is expected to provide the ability to recruit new binding partners to RACK1. Here we show that *Arabidopsis* RACK1A protein homo-dimerizes and tyrosine phosphorylation at key residue regulates the dimerization event to mediate UV-B stress signaling pathway.

## Materials and Methods

### Yeast Split-Ubiquitin Assay

RACK1A homo-dimerization was analyzed using the yeast (*Saccharomyces cerevisiae*) split-ubiquitin system (Stagljar et al., 1998). Gateway compatible plasmid bait – pMet-KZ::GWY cassette-Cub-URA3-CYC1 (His) and prey- pCup-NuI-GWY cassette-CYC1 (Trp) vectors and yeast cells were kindly provided by Dr. Imre Somssich (Max-Planck Institute, Germany). Wild type Yeast strain JD53 (*MATα his3-Δ200 leu2-3112 lys2-801 trp1-Δ63 ura3-52*) were maintained in the YEPD medium as described by Johnsson and Varshavsky (1994). The PCR amplified full length *RACK1A* or the mutant *Y248F-RACK1A* DNA without the stop codon was cloned in the respective bait and prey vectors and were electroporated in the JD53 yeast cells (Biorad-Gene Pulser). Electroporation was performed by using the set program with a voltage of 1500V, 25 μF capacitance, and 200 Ω resistance. Electroporation was done for 5.1 ms by using 2 mm cuvette.

The transformed yeast cells with bait were selected on the SD-His selection plates while the prey vector maintaining yeast cells were selected on SD-Trp selection plates. SD plates were prepared with yeast nitrogen base and drop out media (HTUL) supplemented with the required amino acids except the selection marker. After assaying for the stability of the bait and the prey by their ability to grow on the selection plates, respective bait containing yeast cells were electroporated with the prey constructs and the co-transformed yeast cells were selected on the SD-HT selection plates. The transformants were streaked on minimal medium containing 5-fluoroorotic acid (5-FOA; 0.1%) and were incubated at 30^o^C for 4 days. The wild type RACK1A bait and prey maintaining yeast cells was named as AA while the Y248F-RACK1A bait and prey maintaining yeast cells were named as YY. The empty bait and prey maintaining yeast cells were named as EE.

### BiFC Analysis

Plasmids for BiFC analysis were kindly provided by Dr. Stanton Gelvin of Purdue University. PCR-amplified coding regions of wild type *RACK1A* or the mutant *Y248F-RACK1A* were introduced into the *pCR8/GW/TOPO* entry vector (Invitrogen, Carlsbad, CA, USA). Entry clones were maintained in the *Escherichia coli TOP10* strain, in LB medium supplemented with spectinomycin (50 μg/ml). Following manufacturer’s instruction for gateway cloning, the RACK1A coding sequences were cloned in the respective (pSAT5-DEST-cEYFP-C1(B) (pE3130)) and (pSAT4-DEST-nEYFP-C1 (pE3136) destination vectors). The destination vectors were maintained in *DH5alpha E. coli* strain on LB plates supplemented with 100 μmg/ml ampicillin. Transient gene expression in onion epidermal cells was performed using a High Efficiency Helios Gene Gun System (Bio-Rad) according to the published protocol (Hollender and Liu, 2010). After bombardment, the onion peels were incubated for 24 h on Murashige and Skoog plates in the dark. Images were obtained by using a Nikon EZ-C1 confocal microscope. The argon (488 nm) laser with appropriate emission filters was used for the visualization of FITC. The UV excitation (405 nm) was used to view the DAPI staining.

### Site Directed Mutagenesis of RACK1A

The sequence of the #1 PCR primer for constructing the Y248F substitution was: 5′-CAGTCCCAACAGGTTCTGGCTCTGTGCT-3′ and the #2 primer sequence was 5′-CAGCACAGAGCCAGAACCTGTTGGGACTG-3′. The site directed mutagenesis experiment was done using the QuickChange kit from the Stratagene following the manufacturer’s instructions. Briefly, PCR was carried out in a 50 μl mixture using 50 ng template plasmid DNA containing full length *RACK1A DNA* (*PCR8/GW/TOPO*), 125 ng of each primer, 10 nmol of dNTPs, 2.5 U of cloned *Pfu* DNA polymerase (Stratagene) in 1.0X *Pfu* polymerase reaction buffer. The thermal cycler was programmed for single base pair substitution as follows: pre-incubation at 95°C for 30 s for initial denaturation (one cycle) and then initial 15 cycles at 95°C for 30 s, 55°C for 30 s and 65°C for 4 min.

One microliter (20 U) of *Dpn*I enzyme was added to the sample (50 μl), and incubated at 37°C for 1 h. Two microliters of the final sample was used to transform competent *E. coli* cells [XL1-Blue Supercompetent Cells) by heat shock method]. The transformants were selected on an LB plate with ampicillin. The mutation is confirmed by plasmid DNA sequencing (Genwiz, Germantown, MD, USA).

### Protein Isolation

Yeast cells were grown overnight at 30°C liquid media, then cells were collected by centrifugation (5000 rpm) and protein extraction was done with yeast extraction buffer YPER (Thermo Scientific, Rockford, IL, USA) containing protease inhibitor, Tyrosine Phosphatase inhibitor (Santacruz Biotechnology, CA, USA) and 20 μM of *N*-ethylmalemide (Sigma–Aldrich, St. Louis, MO, USA) to protect isolated proteins from proteases. Concentration of all proteins was measured using Biorad’s Bradford dye following the manufacturer’s instructions.

### SDS-PAGE and Western Blot

Protein samples were resolved by 10% SDS-PAGE gel. After separation, the proteins were transferred to nitrocellulose membranes. The membranes were incubated in blocking buffer (3% skim milk in TBST) for 30 min then washed three times and incubated in Tris Buffer Saline Tween20 (TBST) buffer for overnight at 4°C containing anti RACK1A antibody raised against the full length *Arabidopsis* RACK1A protein (Abcam, Cambridge, MA, USA). To detect phosphorylated Y248 residue, a separate antibody was raised using the epitope: FSPNR{pTYR}WLCAATEH (Genscript, Piscataway, NJ, USA). The membrane was washed three times with TBST and incubated with a horseradish peroxidase (HRP)-conjugated secondary anti-rabbit antibody (Jackson ImmunoResearch, Westgrove, PA, USA). The proteins were visualized by using chemiluminescence reagent (Biorad, Hercules, CA, USA) by exposing to X-ray film (ISC BioExpress, Kaysville, UT, USA). In addition, ChemiDoc^TM^ XRS^+^ gel imaging system (BioRad, Hercules, CA, USA) coupled with a Charge Coupled Device camera is used to take the digital image of the Western blot. The Quantity one software (Biorad) is used to acquire the image and the image is processed by the PhotoShop CS2 software (San Jose, CA, USA).

### Live and Dead Cell Assay

Yeast cells were treated with UV-B lights (American Ultraviolet Co., Lebanon, IN, USA) for 30 min from 12 inches above the plates and a set of all cells were kept under bright light as control. Both treated and non-treated cells were stained with FUN^®^ 1 (Invitrogen, Carlsbad, CA, USA). Cell viabilities were determined by the metabolic activity of fungal cells by fluorescence microscopy. The metabolic activities from the live cells converts the yellow–green–fluorescent intracellular staining of FUN 1 into red–orange intravacuolar structures. Only metabolically active cells are marked clearly with fluorescent intravacuolar structures, while dead cells exhibit extremely bright, diffuse, green–yellow fluorescence. Cells with intact membranes but with little or no metabolic activity have diffuse green cytoplasmic fluorescence and lack fluorescent intravacuolar bodies. The treated cells were visualized under an epifluorescence microsope using 488/530 excitation/emission filter (Nikon, Axiovert 40 CFL).

### Serial Dilution of Treated and Non-Treated Cells on Plate

Serial dilution experiments were performed to measure difference of growth in UV-B treated and non-treated cells. Cells were grown overnight at 30 C by vigorous shaking. The cell density was adjusted to 6 × 10^7^cells/ml (OD_600_ = 3) with media, and the cultures were serially diluted by a factor of 10. For each dilution, 2 μl of cell solution was plated on the indicated selection plates and incubated over 72 h at 30^o^C.

## Results

### RACK1A Proteins Homo-dimerize

**FIGURE 1:**
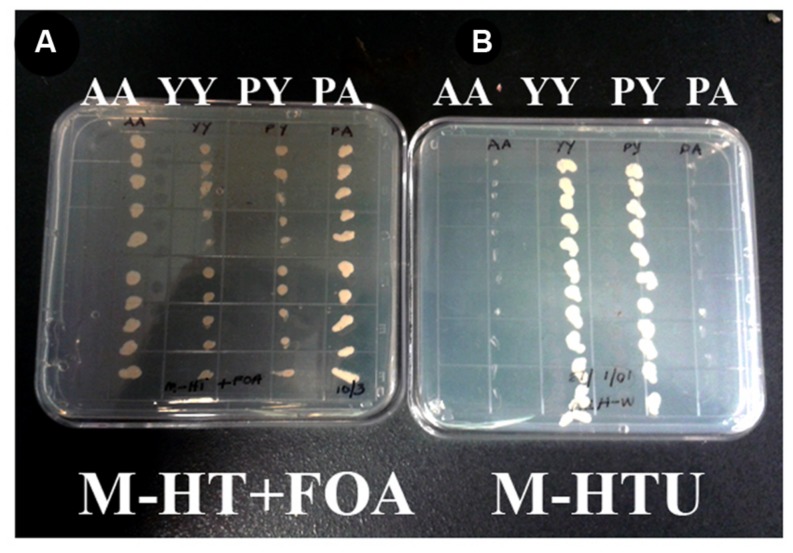
**RACK1 proteins are capable of interacting with each other as shown by the proximity sensor- Split ubiquitin based assay.** Full-length cDNAs from *RACK1A*, *and from Y248F-RACK1A were used to* construct the baits and preys. **(A)** Wild type bait and prey maintaining yeast cells are termed as AA and mutant bait and prey maintaining yeast cells are termed as YY. Growth of AA but not YY on FOA (5-fluoroorotic acid) containing plates confirm interaction. Known RACK1A interactor Plastocyanin (AT1G20340) is used as positive control (RACK1A bait and plastocyanin as prey (PA); while mutant Y248F-RACK1A fails to interact with wildtype plastocyanin (PY). **(B)** Same AA, YY, PY, and PA cells were grown on selective plates without uracil supplement. Interacting proteins (AA and PA) fails to grow on the plate as the URA3 reporter is degraded upon interaction; whereas YY and PY could grow as non-interaction allows to have the reporter URA3 intact that allows them to grow even without uracil supplement.

WD40-mediated homo- and hetero-dimerization of RACK1 has been shown in metazoans to form transient signaling complex by increasing surface area for interaction (Thornton et al., 2004). Blade six in RACK1, in particular emerges to be the major docking station of RACK1 protein, possibly because it undergoes tyrosine phosphorylation followed by conformational changes (Sklan et al., 2006). Within the blade six, residue Y248 has been implicated in diverse physiological processes specifically in RACK1A protein’s interaction with other proteins (Kundu et al., 2013). To understand the role of RACK1A tyrosine phosphorylation on the Y248 residue in terms of homo-dimerization and whether the post-translation modification of the residue has any physiological role, yeast cells were transfected with *Arabidopsis* WT RACK1A split-ubiquitin based bait and prey vectors (pMKZ-RACK1A:NUI-RACK1A abbreviated as AA) or with mutant (Y248F) bait and prey vectors (pMKZ-RACK1A-Y248F:NUI-RACK1A-Y248F abbreviated as YY). The split-ubiquitin method is based on the ability of Nub and Cub, the N- and C-terminal halves of ubiquitin fused to a bait or to a prey separately, to assemble into a quasi-native ubiquitin (Ub) (Thaminy et al., 2003). Ub specific proteases recognize the reconstituted Ub, though not its halves, and cleave the Ub moiety off of a reporter protein, which has been linked to the C-terminus of Cub. The release of the reporter serves as readout indicating interactions. In case of interaction, the reporter URA3 is liberated and hence interacting clones are not able to grow on plates lacking uracil (M-HTU) (**Figure 1B**). The same interacting clones are able to grow on counter selection agent 5-flouoroorotic acid (FOA) containing plates (**Figure 1A**) indicating that WT RACK1A proteins were able to interact with each other. The lack of growth of the YY cells clearly indicates that mutagenesis of Y248F residue abolished the homo-dimerization of RACK1A – implicating Y248 as a target for manipulating RACK1A function. A known RACK1A interactor- plastocyanin protein was used as positive control for FOA selection which shows clear growth on FOA containing plates when co-transfected with the wild type RACK1A (PA) (**Figure 1A**) but no growth when co-transfected with the mutant RACK1A-Y248F (PY) (**Figure 1A**). Further control evidence is provided when the known RACK1A interactors –PA containing yeast cells failed to grow on selection media lacking uracil as the interaction between the two proteins led to the degradation of URA3 reporter protein (**Figure 1B**) but non-interacting PY cells could grow in the absence of uracil in the plate (**Figure 1B**). In the same token the interacting protein (AA) containing cells were not able grow on media lacking uracil while non-interacting YY cells were able to grow on the uracil lacking plates (**Figure 1B**).

**FIGURE 2:**
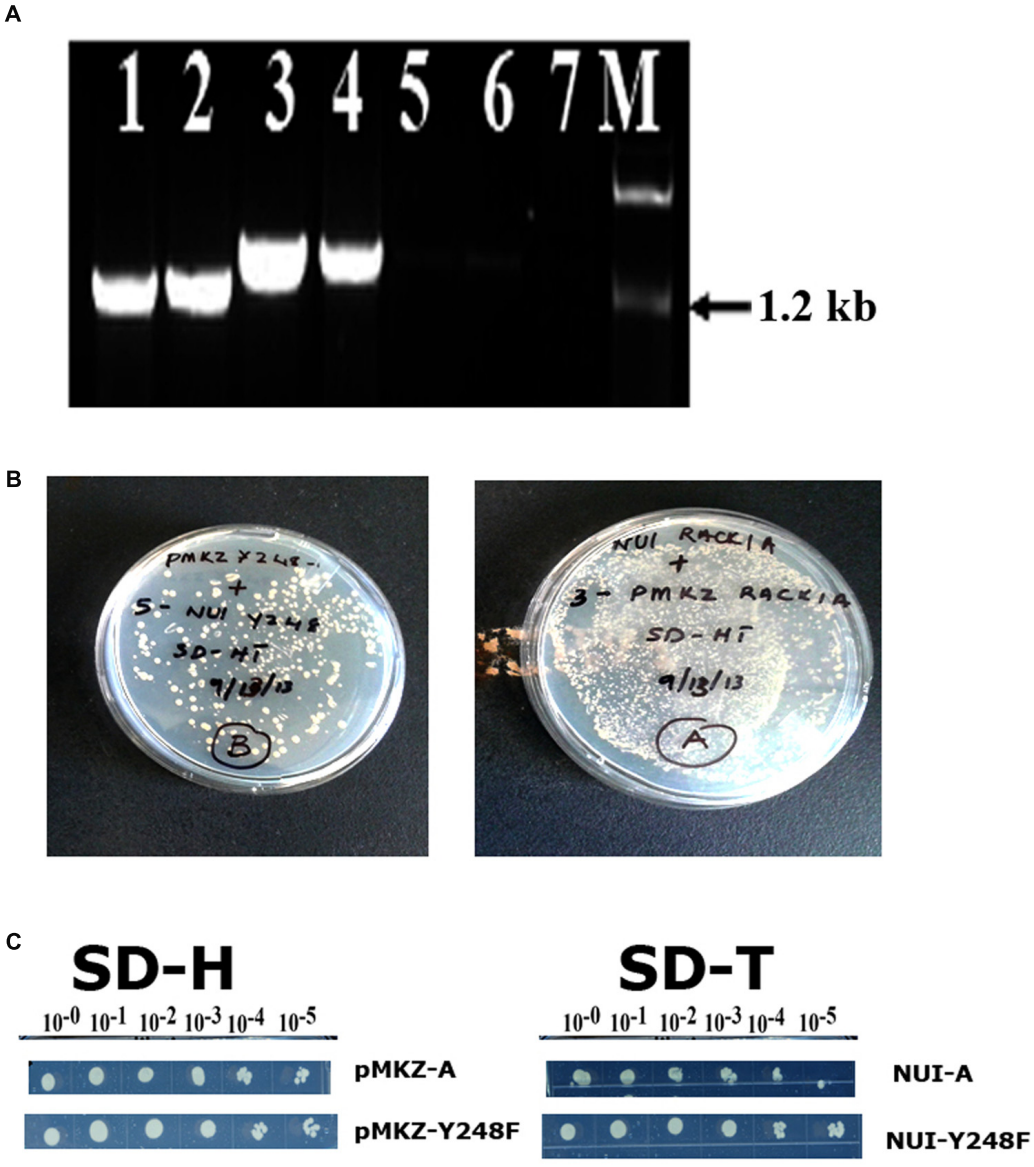
**Stable expression of bait and prey constructs.** **(A)** PCR amplification of bait construct using RACK1A and bait specific (lane 1- AA; lane 2- YY) or RACK1A and prey specific (lane 3- AA and lane 4- YY) primers. The empty vector containing EE cells did not show any amplification from either primer pairs (lane 5 and lane 6). No template control (lane 7) did not amplify any band. **(B)** Yeast cells maintaining both the bait and prey constructs [pMKZ-RACK1A:NUI-RACK1A (abbreviated as AA) and pMKZ- RACK1A-Y248F:NUI-RACK1A-Y248F (abbreviated as YY)] are able to grow of media lacking both the selection markers (minus histidine and tryptophan)- confirming stable expression of the constructs in the AA and YY yeast cells. **(C)** Serial dilution of the individual prey constructs (pMKZ-RACK1A (left upper) and pMKZ-RACK1A-Y248F (left lower) are able to grow on the SD-H selection plates while the individual bait constructs (NUI-RACK1A (right upper) and NUI-RACK1A-Y248F (right lower) are able to grow on the bait specific selection plates (SD-T).

In order to ascertain that the baits were present in the construct and was in the stable condition, several control experiments were also undertaken (**Figure 2**). The presence of *RACK1A* DNA in the yeast cells was also ascertained in a PCR reaction using RACK1A specific and bait (lane 1- AA, lane 2- YY) or prey specific (lane 3- AA, lane 4- YY) PCR primers. The empty vector containing EE cells did not show any amplification from either primer pairs (lane 5 and 6). No template control (lane 7) was also used with bait and RACK1A specific primer. To confirm that transfected yeast cells stably expressed the bait (pMKZA-RACK1A or pMKZ-RACK1A-Y248F) and the prey constructs (NUI-RACK1A or NUI-RACK1A-Y248F), the yeast cells harboring both the prey and the bait constructs were grown on media lacking the marker amino acids for the bait and the prey(SD-HT) and the **Figure 2B** clearly shows that yeast cells harboring either the wild type (AA) or the mutant (YY) constructs were able to grow on the double selection plates of SD-HT. To further confirm the stability, a serial dilutions of both the bait constructs (pMKZA-RACK1A and pMKZ-RACK1A-Y248F) were plated on the SD-H selection plates and the cells were able to grow on the selection plates (**Figure 2C**, left). Similar serial dilution assay were used to check the stability of the prey constructs as well. The serially diluted individual prey constructs (NUI-RACK1A and NUI-RACK1A-Y248F) were also able to grow on the prey specific selection plate SD-T (**Figure 2C**, right). To confirm that transfected yeast cells stably expressed both the bait and prey constructs, the cells were grown on media lacking the marker amino acids for the bait and the prey (SD-HT: Histidine and Tryptophan) and the **Figure 2B** clearly shows that both the AA and YY cells were able to survive on the plate lacking both the selections (Histidine and Tryptophan) SD-HT plates. With all these controls, it is concluded that both the baits and prey from the WT and mutant constructs are stably expressed in the transformed yeast cells designated as AA and YY cells. Further evidence of the expression was obtained through the use of western blot as described in **Figure 4B**.

### RACK1A Proteins Homo-dimerize *In Vivo*

**FIGURE 3:**
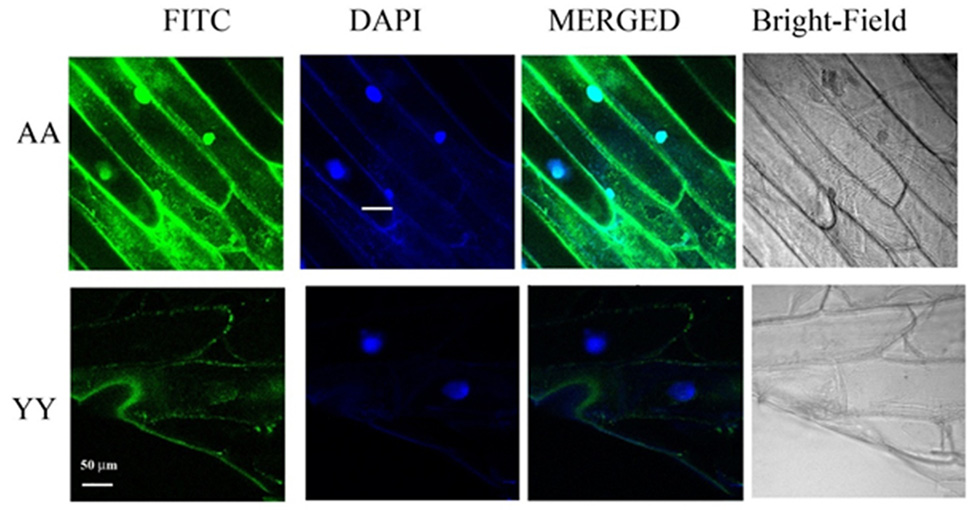
**BiFC assays confirm RACK1A homo-dimerization *in vivo*.** BiFC analysis shows homo-dimerization of RACK1A in the nucleus and plasma membrane of onion epidermal strips. Onion epidermal cells bombarded with nYFP-RACK1A and cYFP-RACK1A constructs (AA) shows YFP fluorescence through fluorescence complementation while cells bombarded with the nYFP-Y248F-RACK1A and cYFP-Y248F-RACK1A (YY) constructs failed to show any fluorescence complementation.

**FIGURE 4:**
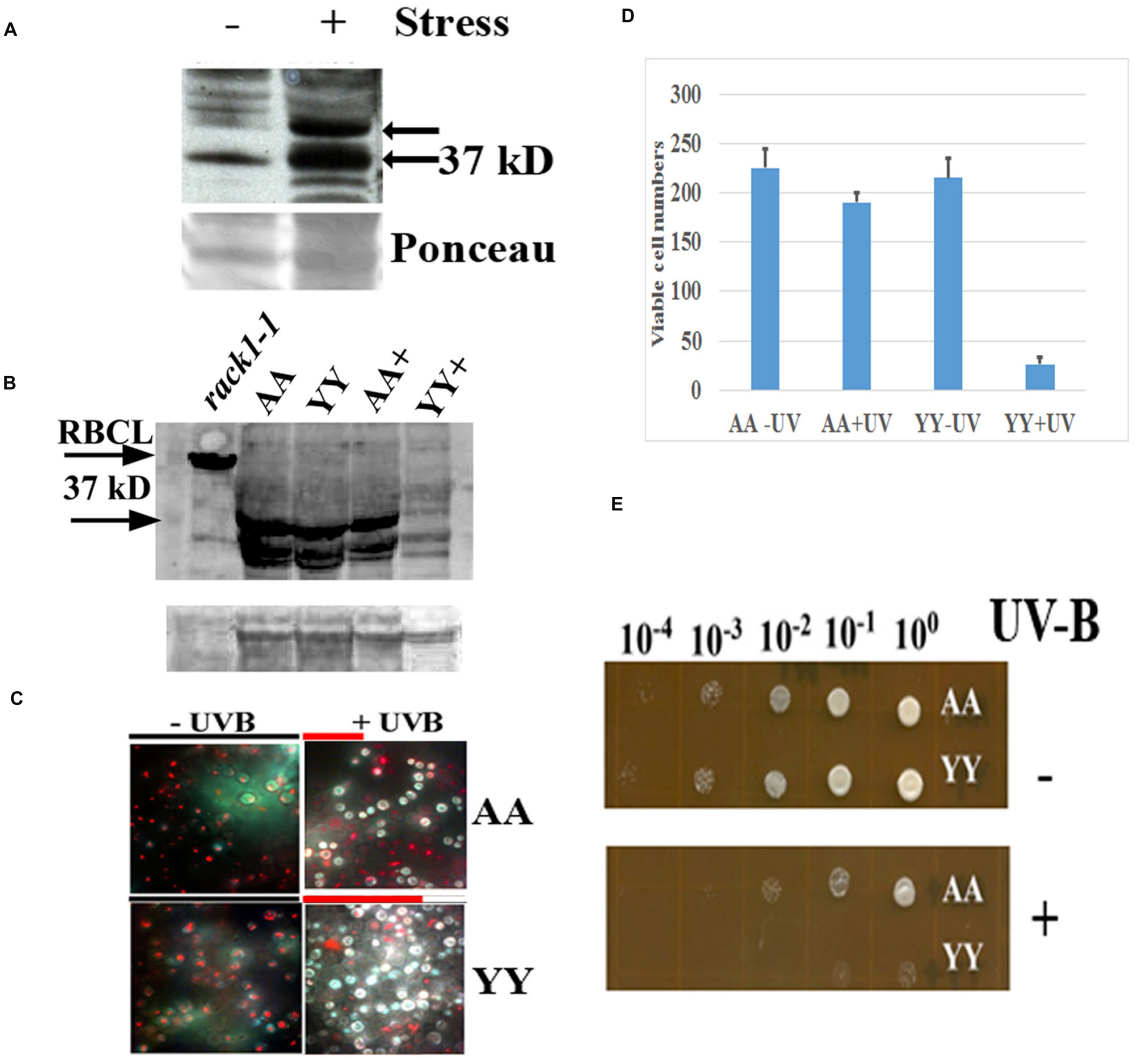
**Physiological effect of RACK1A homo-dimerization.** **(A)** Phosphorylation of RACK1A Y248 residue under heat stress. Anti-pY248-RACK1A antibody is raised by adsorbing against the RACK1B and RACK1C peptides to ensure that the antibody would only recognize the Y248 phosphorylated RACK1A protein (Creative Diagnostics, Shirley, NY, USA). Y248 phosphorylation is enhanced in the presence of high heat (37°C) compared to the ambient heat (22°C). *Arabidopsis* plants (3 weeks-old) were incubated for 3 h at the indicated temperatures with constant light and as loading control, the ponceau stained blot image is presented at the bottom of the panel. **(B)** Stable expression of RACK1A protein is evident under the UV-B exposed (45 min) AA cells but UV-B exposed YY cells show instable RACK1A expression. Full length anti-RACK1A antibody detects both the RACK1A proteins in the bait and in the prey vectors. The lysates from the rack1a-1 knock-out *Arabidopsis* plants were used as negative control which did not show any 37 kD band but did show the Rubisco large subunit (RbcL ∼55 kD) band implying loading of lysates in the lane. Bands from non-specific binding lanes are used as loading control (lower lane). Note that *Arabidopsis* lysate did not show the non-specific band. The image is acquired using a ChemiDoc^TM^ XRS^+^ gel documentation system (Biorad, Hercules, CA, USA). **(C)** AA yeast cells can withstand UV-B induced stress conditions. AA and YY yeast cells were grown on M-HT medium at 30°C overnight, then exposed to UV-B lights (2.0 mW/cm^2^) for 45 min. As control, another set were treated to ambient light condition. An equal aliquot of the UV-B treated and non-treated cells were grown in M-HT medium for 48 h at 30°C. Yeast cell death is evaluated with the yeast viability kit (Invitrogen Inc. CA). AA cells show increased resistance to UV-B radiation induced cell death, whereas YY cells show susceptible phenotype (right). The non-treated AA and YY cells did not show significant cell death (left). The red lines above the + panels represents relative level of cell survival. **(D)** Quantitative evaluation of cell death under UV-B stress. Viable cells from three independent experiments as described in panel C were counted (total 300 cells). The mean is presented in the graph. The standard error of the mean from each treatment is also computed and is presented as bar on each mean value. **(E)** UV-B treated and non-treated AA and YY cells as in panel C were serial diluted to measure the growth potential on M-HT plates. AA cells as opposed to the YY cells show resistance to growth inhibition (lower). Non-treated AA and YY cells did not show any growth inhibition (upper).

Whether the homo-dimerization of RACK1A protein as evident from the split-ubiquitin based assay also occurs *in vivo*, YFP fluorescence based Bimolecular Fluorescence Complementation (BiFC) assay was conducted. As the BiFC allows researchers to visualize protein–protein interactions in living cells, the assay not only provides evidence for protein-interaction, it also shows the cellular sites of interaction to provide additional functional information. As can be seen from the **Figure 3**, YFP fluorescence signal was observed in the onion epidermis cells co-expressing nYFP-RACK1A and cYFP-RACK1A constructs (AA) while cells co-expressing nYFP-Y248F-RACK1A and cYFP-Y248F-RACK1A (YY) failed to show any fluorescence. The empty vectors were also used as a control which also did not show any complementation of YFP fluorescence (data not shown). However, it remains to be ascertained whether the lack of fluorescence from the YY expressing tissues was due to the instability of the mutant constructs. In the light of stable expression of the mutant constructs in the transfected yeast cells (**Figures 2B,C** and **4B**), it is reasonable to expect that the construct instability had not resulted in the lack of fluorescence in the YY expressing tissues. Merging of the FITC and the DAPI channel images clearly indicates that majority of the homo-dimerization event occurs in the plasma membrane and in the nucleus. It remains to be determined whether dimerization in the nucleus allows the protein to regulate gene expression from target proteins.

### Physiological Role of RACK1A Homo-dimerization

In an earlier experiments, high heat condition indicated that *Arabidopsis* RACK1A Y248 residue is a major target in the stress response pathway (**Figure 4A**). The antibody raised to detect the Y248 phosphorylation was clearly able to show that during high heat condition, the Y248 residue does get phosphorylated. The inability of the anti-RACK1A-pY248 antibody to detect Y248 phosphorylation event in the yeast cells led us to use the full length *Arabidopsis* anti-RACK1A antibody to evaluate the physiological role of the RACK1A homo-dimerization event. In addition, lack of suitable conditions to mimic comparable high heat condition in yeast cells growing in liquid media, we have settled on the study of oxidative stress condition response in these experimental yeast lines. Oxidative stress was generated through the exposure to low level of UV-B (290–320 nm: 2.0 mW/cm^2^) light for 30 min (Mackerness et al., 2001; Chen et al., 2012). Yeast cells harboring the wild-type RACK1A termed as AA (pMKZ-RACK1A with NUI-RACK1A) and mutant (pMKZ-RACK1A-Y248F with NUI-RACK1A-Y248F) bait and prey constructs (YY) were treated with the indicated intensity of UV-B lights. As a control, similar densities of cells were kept for the same amount of time under ambient lab light. UV-B treated cells and non-treated cells were serial diluted and plated on M-HT plates and compared to the growth of the YY yeast cells, the AA yeast cells appeared to be highly resistant to the UV-B induced cell death (**Figure 4C**). Similar results were obtained when same sets of cells were allowed to grow in liquid culture over-night. Only AA yeast cells could grow whereas the YY yeast cells failed to grow after the UV-B treatment (data not shown) -indicating that RACK1A homo-dimerization allows the cells to withstand UV-B induced stress conditions.

To ascertain whether the UV-B stress induced resistance results in more cell survival, yeast live and dead cell assay (Invitrogen, Carlsbad, CA, USA) was utilized on the designated cell types (AA and YY). The respective cells were exposed to UV-B radiation for 45 min as well as ambient light with same experimental condition, then stained with yellow green FUN 1 dye that is converted to red-orange intra-vacuolar structures in metabolically active cells. The dead cells failed to produce the red-orange intra-vacuolar structures. As can be seen from the **Figure 4D**, the UV-B treated AA yeast cells showed increased resistant to cell death compared to the UV-B treated YY cells. Quantification of viable cells after the UV-B treatment confirmed the increased resistance to UV-B radiation induced cell death in the yeast cells expressing homo-dimerized RACK1A proteins; whereas, the Y248F mutation of the RACK1A led to the disruption of the homo-dimerization of the RACK1A which presumably led to the UV-B induced cell death in the YY cells (**Figure 4D**). This observation was also confirmed when the UV-B treated and non-treated serially diluted AA and YY cells were grown on the M-HT plates (**Figure 4E**). While the non-treated AA and YY cells were able to grow in almost all the dilutions equally (**Figure 4E**, upper panel), the UV-B treated AA cells, compared to the UV-B treated YY cells, could grow almost up to the 100 fold dilution (**Figure 4E**, lower panel). This result indicates that the homo-dimerization of RACK1A can potentially allow the yeast cells to withstand UV-B induced cell death most likely by activating ROS detoxifying enzymes. It also appeared that without any exposure to the UV-B radiation, both the AA and YY cells expressed the RACK1A proteins- indicating the stable expression of the bait and prey proteins from the Wildtype RACK1A and the Y248F mutant transfected yeast cells (**Figure 4B**). The lysates from the yeast cells as treated in **Figure 4D** were run on a SDS-PAGE gel and transferred to a PVDF membrane and the expression of the bait and prey constructs were evaluated with a full length *Arabidopsis* anti-RACK1A antibody (Abcam, Cambridge, MA, USA). As can be seen from the **Figure 4B**, both the prey and the bait constructs in the AA and YY cells expressed the expected ∼37 kD bands under no UV-B treatment condition. While the AA cells were able to maintain the expression of the prey and bait proteins, albeit at a lower level, with the exposure to the UV-B radiation, the YY cells completely abolished the expression of RACK1A proteins from the bait and prey constructs (**Figure 4B**, lane YY^+^). This indicates that the loss of RACK1A expressions in the YY cells can be attributed to the increased susceptibility to the UV-B induced radiation under the experimental conditions in the yeast cells. As a negative control, lysates from the leaves of 2 weeks-old RACK1A knock-out *Arabidopsis* plants (*rack1a1-1*) were used and the antibody failed to detect any significant band near the expected 37 kD size (**Figure 4B**, lower arrow) implying that the antibody did detect the RACK1A proteins in the yeast lysates. Even though the *rack1a-1* plants maintain the other two copies of the *RACK1* genes, it is known that the RACK1A is the predominant isoform of the RACK1 proteins (Guo and Chen, 2008). As loading control for the *Arabidopsis* lysates, the abundantly expressed RbcL protein in the leaf tissues was used (**Figure 4B**, upper arrow) and for the yeast cells, a non-specific band on the same blot is used as loading control (**Figure 4B**, lower).

## Discussions

Role of RACK1 in diverse signaling processes has been implicated and well established, yet the molecular mechanisms of dimerization and their role in abiotic stress signaling, especially oxidative stress like UV-B radiation, are largely unknown. Here we show evidence that RACK1A proteins homo-dimerize and the tyrosine residue at the 248 position play a central role in the homo-dimerization process. The homo-dimerized RACK1A proteins were able to withstand UV-B induced oxidative stress in yeast cells.

Mutagenesis work on the key tyrosine residues implicated Tyr228 and Tyr246 phosphorylation to increased binding affinities of metazoan RACK1 proteins (Chang et al., 2001). Interestingly, the two proposed tyrosine phosphorylation sites of human RACK1 are conserved in *A*. *thaliana* RACK1A (Tyr230 and Tyr248). Though no *bona fide* tyrosine kinase has been identified in plants, tyrosine phosphorylation, and dephosphorylation have been documented to regulate growth and development in seeds, hypocotyls, and roots in *A. thaliana* (Reyes et al., 2006; Hussain et al., 2007; Yemets et al., 2008). It is hypothesized that tyrosine phosphorylation in plants as well as in yeast is carried out by dual-specificity serine/threonine/tyrosine (STY) protein kinases (Stern et al., 1991; Rudrabhatla et al., 2006). *Bona fide* tyrosine-specific protein phosphatases do exist in *Arabidopsis* (Xu et al., 1998; Luan, 2002, 2003). Though it is accepted that plants and animals do not share mechanistic features of tyrosine phosphorylation, a recent proteome wide mapping of *in vivo* phosphorylation sites in *Arabidopsis* has identified 2172 sites, of which 94 sites were identified as tyrosine residues (Sugiyama et al., 2008). The study estimated the relative abundances of pY in *Arabidopsis* as 4.3%- a number very close to the 1.8–6.0% of pY estimated in human cells (Sugiyama et al., 2008). Recently it has been shown that phosphorylation of the RACK1A protein at two residues: Ser-122 and Thr-162 by an atypical serine (Ser)/threonine (Thr) protein kinase WNK8 (WITH NO LYSINE8) negatively regulates RACK1A function in the glucose responsiveness pathway by influencing the stability of the protein (Urano et al., 2015). Here we show that Y248 residue phosphorylation also influences the stability of the RACK1A protein under oxidative stress condition. It remains to be seen whether these two phosphorylation events are coupled to determine the stability of the protein.

Though majority of RACK1 studies were reported using monomeric form of the protein, dimeric form of the proteins are also reported in the literature. For example, it is reported that dimerized RACK1 can concomitantly bind to *N*-methyl-D-aspartate (NMDA) receptor and its cognate regulator- the Fyn kinase (Thornton et al., 2004). Liu et al. (2007) found the dimerization of RACK1 is needed for the oxygen-independent degradation of hypoxia-inducible factor (HIF-1). In addition, like many other WD40 repeat proteins, RACK1 interacts with closely similar G-protein beta subunit through the WD40 repeat domains to regulate the nuclear localization of RACK1 (Chen et al., 2004). The first structure of a RACK1 homolog in yeast reveals that yRACK1 dimerizes through blade 4—based on a mechanism that may be conserved among RACK1 proteins. These interactions may enable additional binding or expose hidden binding sites in RACK1. In scanning for any solvent exposed tyrosine residue near the WD repeat 6, the *Arabidopsis* RACK1A crystal structure clearly shows that the side chain of Tyr248 is located at the end of the loop connecting β-strands A and B of blade 6 and is fully exposed to the solvent, with the putative phosphate acceptor hydroxyl pointing away from the protein, easily accessible for modification (Ullah et al., 2008). Using WT *Arabidopsis* RACK1A as bait, Kundu et al. (2013) reported 97 interacting proteins; however, when the mutant RACK1A-Y248F was used as bait, no interacting proteins was identified- indicating the key role this tyrosine residue play in mediating protein–protein interactions. In non-plant systems, mutagenesis work has identified the conserved Tyr228 and Tyr246 as potential phosphorylation sites and suggested a correlation between enhanced tyrosine phosphorylation of RACK1 and binding of RACK1 to Src (Chang et al., 2001). RACK1 binding to heterotrimeric Gβγ results in translocation from the cytosol to the plasma membrane, suggesting that this role of the interaction is to alter the cellular localization of RACK1 and constitute scaffolds for the assembly of multiple signaling protein complexes (Chen et al., 2008).

Though the physiological role of this dimerization is still unclear, in the regulation process of the NMDA receptor by Fyn, RACK1 dimerization is required to bring the two interacting partners in close contact (Thornton et al., 2004). Indeed, the NMDA receptor and the Fyn kinase share a common binding site on the RACK1 monomer. As a simple consequence, they can only interact simultaneously with a RACK1 homodimer. Similarly, the oxygen dependent degradation of HIF-1 requires RACK1 dimerization to allow Elongin C to be recruited in the vicinity of HIF 1 (Liu et al., 2007). In both cases, the RACK1 dimer serves as a bridge between two interacting molecules to allow a specific process to occur. It is possible that RACK1 dimerization allows exposing a new surface of the protein, buried within the propeller core in the monomeric form. RACK1 dimerization may therefore present a double advantage. Through this duplication mechanism, it can allow bringing into close contact two partners that interact on the same surface of RACK1 and would consequently not be able to interact together through a monomeric RACK1. In addition, it may also allow recruiting novel interacting partners via this newly exposed region that remains inaccessible within the monomer. The Y248 residue can now serve as a target to modulate RACK1A function in crops where the protein is known to negatively regulate the stress hormone abscisic acid signaling pathways to regulate few several environmental stress signaling pathways including the drought stress signaling pathways (Ullah et al., 2008).

RACK1 dimerization allows exposing an entirely new surface of the protein to the solvent, and this region becomes potentially accessible to new interacting partners. We therefore speculate that this may be one of the advantages of homodimer formation and thereby, RACK1 could increase its panel of binding partners under specific physiological conditions.

## Author Contributions

HU: planned the experiments and contributed significantly in writing the manuscripts. MS: conducted the experiments. NK: conducted the experiments. DS: conducted few of the experiments.

## Conflict of Interest Statement

The authors declare that the research was conducted in the absence of any commercial or financial relationships that could be construed as a potential conflict of interest.
